# Supplementary material for: From Brain Lobes to Neurons: Navigating the Brain Using Advanced 3D Modeling and Visualization Tools
Source: J Imaging. 2025 Sep 1;11(9):298. doi: 10.3390/jimaging11090298 (PMC12470745; doi:10.3390/jimaging11090298)
Supplement: Supplementary file 1 [file jimaging-11-00298-s001.zip › jimaging-3786501-supplementary.pdf]

Supplementary material

Table S1. Software and parameter settings used in this study.

	Application	Action	Parameters
1	www.imalos.com	Download	Download MRI Scans as .png file
2	Adobe Photoshop	Selecting Cerebrum	• 'Magic Wand Selection' tool<br>• Sample Size: Point Sample<br>• Tolerance: 30
3	Fiji	Image Importing	• File > Import > 'Image Sequence', Scale 100%, Sort Numerically
4	Fiji	Image Stacking	• Image > Stacks > 3D Project<br>o Project Method: Brightest Point<br>o Axis: Y Axis<br>o Slice Spacing (pixels): 1<br>o Interpolate: On<br>o Remaining parameters as default<br>• Plugin > 3D Viewer<br>o Display > Surface<br>o Color > White<br>o Threshold : 50<br>o Resampling Factor : 4<br>• File > Export Surfaces > STL (binary) / .obj
6	Rhino 6	Verify Model	• Import STL File<br>• View > Shaded View<br>• Bounding Box > Scale 1D of Height<br>• Export to .obj
7	MeshLab	Clean/Edit	• Import .obj file<br>• Filters > Cleaning and Repairing > Repair non Manifold Edges by removing faces<br>• Filters > Cleaning and Repairing > Repair non Manifold Edges by splitting vertices<br>• Filters > Cleaning and Repairing > Remove duplicate faces<br>• Filters > Cleaning and Repairing > Remove Isolated Pieces<br>• Filters > Remeshing, Simplifications and Reconstruction > Uniform Mesh Resampling. Precision: 0.3, 1. Clean Vertices ON<br>• Filters >Smoothing, Fairing, Deformation > Laplacian Smooth > Smoothing Steps: 4

			• Filters >Remeshing, Simplification and Reconstruction > Remeshing: Isotropic Explicit Remeshing > Target Length (Percent): 0.5 Percent<br>• Filters > Smoothing, fairing and deformation > Taubin Smooth<br>• Filters > Smoothing, Fairing and Deformation > HC Laplacian Smooth > Apply<br>• Filters > Remeshing, Simplification and Reconstruction > Simplification: Quadric Edge Collapse Decimation > preserve boundary of the mesh, preserve normal, preserve, post-simplification cleaning ON<br>• Mesh Optimization<br>• Export Mesh As > .obj file
8	Houdini	Import	Import Brain 3D File to Houdini
9	Houdini	Slicing Brain	• Command ' Knife', select 'Keep: Primitives Below the plane'<br>• Apply key frames from starting point to end point of desired location of the slicing to animate and export to video.<br>• Spot Lighting 1:<br>o Type: Point<br>o Color: FFAE9C<br>o Intensity: 0.794<br>o Exposure: 5.833<br>o Angle: Top Right (transform and move light source accordingly)<br>• Spot Lighting 2:<br>o Type: Point<br>o Color: FFAE9C<br>o Intensity: 0.1<br>o Exposure: 5.833<br>o Angle: Top Center (transform and move light source accordingly)<br>• Spot Lighting 3:<br>o Type: Point<br>o Color: FFAE9C<br>o Intensity: 0.35<br>o Exposure: 5.833<br>o Angle: Top Left (transform and move light source accordingly)<br>• Area Lighting 4:<br>o Type: Grid

			○ Color: FFC37A<br>○ Intensity: 0.16<br>○ Exposure: 5.833<br>○ Angle: Bottom Left (transform and move light source accordingly)<br>• Area Lighting 5:<br>○ Type: Grid<br>○ Color: 525DFF<br>○ Intensity: 0.16<br>○ Exposure: 5.833<br>○ Angle: Bottom Center (transform and move source accordingly)<br>• Area Lighting 6:<br>○ Type: Grid<br>○ Color: 5245FF<br>○ Intensity: 0.3<br>○ Exposure: 5.833<br>○ Angle: Bottom Righ (transform and move light source accordingly)<br>• Area Lighting 7:<br>○ Type: Grid<br>○ Color: 83A7FF<br>○ Intensity: 0.3<br>○ Exposure: 5.833<br>○ Angle: Middle Right (transform and move light source accordingly)<br>• Camera:<br>○ Resolution: 1280, 720<br>○ Pixel Aspect Ratio: 1<br>○ Projection: Perspective<br>○ Focal Length: 50<br>○ Aperture: 41.4<br>○ Near Clipping: 0.1<br>○ Far Clipping 4095.98<br>○ Shutter time: 0.5<br>○ Focus Distance: 6.92<br>○ F-Stop: 5.6
10	Houdini	Export Frames	• Render key frames to .png format with resolution 1280 x 720
11	Houdini	Painting Brain	• Command 'attribwrangle', Run Over: Points<br>• Command 'attribpaint'<br>○ LMB Operation: Paint FG, Shift: Smooth, Ctrl:Paint BG

			o Paint Mode: Over<br>o Shape: Volume<br>o FB Color: as required<br>o BG Color: as required<br>o Opacity: 1<br>o Soft Edge: 0.5<br>o Opacity Pressure: 1<br>o Radius Pressure: 1<br>o Attributes: 1, Attribute Name: Cd, Type: Color<br>• Spot Lighting 1:<br>o Type: Point<br>o Color: FFAE9C<br>o Intensity: 0.794<br>o Exposure: 5.833<br>o Angle: Top Right (transform and move light source accordingly)<br>• Spot Lighting 2:<br>o Type: Point<br>o Color: FFAE9C<br>o Intensity: 0.1<br>o Exposure: 5.833<br>o Angle: Top Center (transform and move light source accordingly)<br>• Spot Lighting 3:<br>o Type: Point<br>o Color: FFAE9C<br>o Intensity: 0.35<br>o Exposure: 5.833<br>o Angle: Top Left (transform and move light source accordingly)<br>• Area Lighting 4:<br>o Type: Grid<br>o Color: FFC37A<br>o Intensity: 0.16<br>o Exposure: 5.833<br>o Angle: Bottom Left (transform and move light source accordingly)<br>• Area Lighting 5:<br>o Type: Grid<br>o Color: 525DFF<br>o Intensity: 0.16<br>o Exposure: 5.833

			o Angle: Bottom Center (transform and move source accordingly)<br>• Area Lighting 6:<br>o Type: Grid<br>o Color: 5245FF<br>o Intensity: 0.3<br>o Exposure: 5.833<br>o Angle: Bottom Righ (transform and move light source accordingly)<br>• Area Lighting 7:<br>o Type: Grid<br>o Color: 83A7FF<br>o Intensity: 0.3<br>o Exposure: 5.833<br>o Angle: Middle Right (transform and move light source accordingly)<br>• Camera:<br>o Resolution: 1280, 720<br>o Pixel Aspect Ratio: 1<br>o Projection: Perspective<br>o Focal Length: 50<br>o Aperture: 41.4<br>o Near Clipping: 0.1<br>o Far Clipping 4095.98<br>o Shutter time: 0.5<br>o Focus Distance: 6.92<br>o F-Stop: 5.6
12	Houdini	Export Frames	• Render key frames to .png format with resolution 1280 x 720
13	Houdini	Animated growth simulation	• Require defining two inputs to a 'solver' node<br>o First input require define the 'seed' by creating a standard 'line'<br>o Second input is the volume in which this line grows, volume imported is the Brain 3D model<br>• Camera:<br>o Resolution: 1280, 720<br>o Pixel Aspect Ratio: 1<br>o Projection: Perspective<br>o Focal Length: 50<br>o Aperture: 41.4<br>o Near Clipping: 0.1<br>o Far Clipping 4095.98

			o Shutter time: 0.5<br>o Focus Distance: 6.92<br>o F-Stop: 5.6
14	Houdini		• Render key frames to .png format with resolution 1280 x 720
15	Cinema4D		• Neuron Material – Fresnel Shader:<br>o R:215, G:97, B:97<br>• Camera<br>o Projection: Perspective<br>o Focal Length: 36mm<br>o Sensor Size: 36mm<br>o Field of View: 53 Degree<br>o Focus Distance: 2000m<br>o Stereoscopic Mode: Mono<br>o White Balance: 6500k<br>o Target: Set target to center of column<br>• Lighting<br>o Color: R:247, G:255, B:153<br>o Intensity: 200%<br>o Type: Omni<br>o Shadow: None
16	Cinema4D	Export	• Render Settings:<br>o Width 1200px, Height 1200px<br>o Resolution: 72 dpi<br>o Film aspect 1:1<br>o Frame Range: as per key frames defined
17	Cinema4D	Cortical Column	• Compose the cortical column with the different layers<br>• Cortical Column Material – Fresnel Shader:<br>o Layer 1: R:177, G:255, B:237<br>o Layer 2: R:255, G:255, B:255<br>o Layer 3: R:177, G:255, B:237<br>o Layer 4: R:97, G162, B:215<br>o Layer 5: R:97, G:215, B:164<br>o Layer 6: R:199, G:97, B:215<br>• Camera:<br>o Projection: Parallel<br>o Zoom: 0.4803<br>o Focus Distance: 2000cm<br>o White Balance(k): 6500k<br>o Stereoscopic Mode: Mono<br>o Target: Set target to center of column

18	Cinema4D		• Render Settings:<br>o Width 1200px, Height 1200px<br>o Resolution: 72 dpi<br>o Film aspect 1:1<br>• Frame Range: as per key frames defined
19	After Effects	Import Media	• Composition Settings:<br>o Width: 1280<br>o Height: 720<br>o Pixel Aspect Ratio: Square Pixels<br>o Frame Rate: 30<br>o Resolution: Full<br>o Duration: 00:01:20:00<br>o Background Color: Black
20	After Effects	Compiling	• Import and stack sequentially
21	After Effects	Animate and Annotate	• Text<br>o Font: Arial<br>o Bold<br>o Size: 60px
22	After Effects	Full Video Rendering	• Add composition to Render Queue<br>o Main options: Format: H.264<br>o Channels: RGB<br>o Depth: Millions of colors<br>o Color premultiplied (matted)<br>• Open adobe media encoder<br>o Output file in .mp4 format

S2: Consort diagram

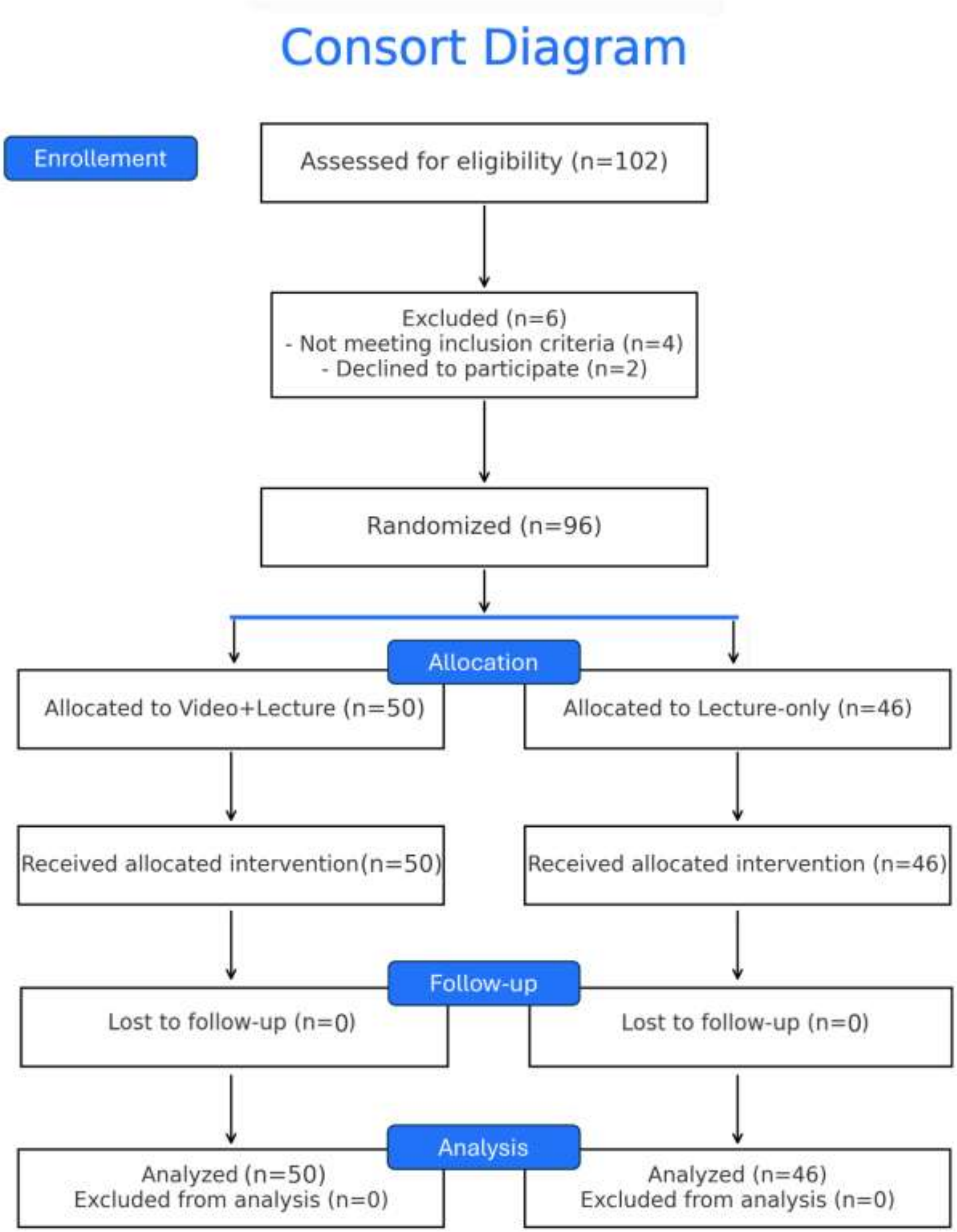

S3: Video link ((https://figshare.com/s/5fd9c530ada5515b7d35)

S4: Learner data (https://zenodo.org/records/16892279)

S5: Pre-post measures (https://zenodo.org/records/16892279)
